# Are ethical norms and current policies still relevant in face of the recent mass terror events?

**DOI:** 10.1186/s13049-016-0304-6

**Published:** 2016-10-04

**Authors:** Tomer Simon, Avishay Goldberg, Bruria Adini

**Affiliations:** 1 Emergency Medicine Department, Recanati School of Community Health Professions, Faculty of Health Sciences, Ben-Gurion University of the Negev, Beersheba, Israel; 2 Department of Health Systems Management, Faculty of Health Sciences, Ben-Gurion University of the Negev, Beer Sheba, Israel; 3 PREPARED Center for Emergency Response Research, Ben Gurion University of the Negev, Beer Sheba, Israel

**Keywords:** Emergency response, Social media, Emergency medical services, Terror, Pre-hospital, Ethics, Privacy, Healthcare policy

## Abstract

The widespread utilization of social media in recent terror attacks in major European cities should raise a “red flag” for the emergency medical response teams. The question arises as to the impact of social media during terror events on the healthcare system. Information was published well before any emergency authority received a distress call or was requested to respond. Photos published at early stages of the attacks, through social media were uncensored, presenting identifiable pictures of victims. Technological advancements of recent years decrease and remove barriers that enable the public to use them as they see fit. These attacks raise ethical considerations for the patients and their rights as they were outsourced from the medical community, into the hands of the public. The healthcare system should leverage social media and its advantages in designing response to terror, but this requires a re-evaluation and introspection into the current emergency response models.

## Main text

The widespread utilization of social media in recent terror attacks of major European cities, including Brussels (March 2016), Paris (November 2015 & June 2016), and Istanbul (June 2016) should raise a “red flag” for the emergency response community, most specifically for medical response teams.

Social media has been researched extensively in regards to emergency management for both natural and manmade emergencies [1, 2]. Major recent disasters, such as the 2010 Haiti earthquake, the 2012 “Super-storm Sandy” or the 2015 Nepal earthquake have all presented social media as the major communication channel between first responders and the public. The question arises as to the impact of social media during terror events, and whether the bi-directional and extremely rapid communication should serve as a wake-up call for the healthcare system concerning ethical norms that up to now have been accepted as important.

During the hours immediately following the Paris and Brussels’ attacks we collected, using TwitterMate (a self-developed system designated to collect tweets from Twitter), the tweets of the public and the emergency responders. During and immediately following the Brussels’ attack, over 400,000 tweets of the public were collected, representing the vast majority of tweets published using the hashtag #Brussels, as well as all tweets (~100,000) posted by Belgium’s emergency and security agencies and the public’s response to them. The analysis of the textual and visual tweets revealed lessons learnt that raise the need to re-formulate some basic beliefs, which are as yet widely accepted principles, and even “sacred” norms, such as patient rights and medical privilege. They also exposed untapped and realizable opportunities to leverage social media, which can improve the ability to respond and manage terror attacks in densely populated areas.

Within a few minutes from the explosions in the Brussels and Istanbul airports, photos from the scene were uploaded to Twitter. These photos were taken on-site, mostly by survivors or bystanders, which provided highly graphic depictions of the injuries, and the overall medical status of the casualties. This information was published well before any emergency authority, outside the airport, received a distress call or was requested to respond.

The photos published at early stages of these terror attacks, through Twitter and other social media were uncensored, presenting identifiable pictures of victims. The privacy of the victims and the dead was grossly invaded without their consent [3, 4]. The general public is unfamiliar with, nor adheres to any ethical guidelines that apply to medical professionals. These photos may reach families and friends prior to receiving any notification and cause unwarranted distress. The technological advancements of recent years decrease and remove barriers that enable the general public to use them as they see fit. The Brussels and Istanbul terror attacks should be a cautionary call for all medical and health institutions as it brings to our attention essential ethical and medical privacy issues that were violated; considerations of the patients and their rights is outsourced from the medical community, into the hands of the general public.

In addition to the need to modify the policy concerning ethics in terror events, social media presents an opportunity to improve the response of medical services by considering the implementation of the following actions.

**Fig. 1:**
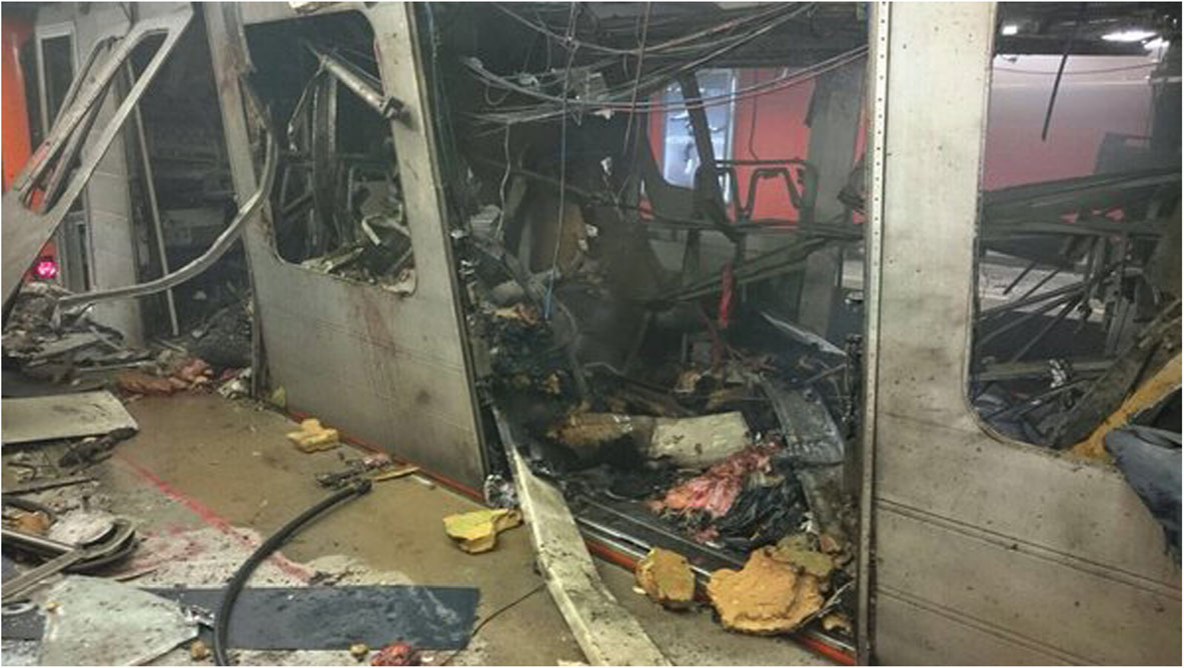
Photo taken minutes after the explosion at the Brussels Metro (taken from Twitter)

The photos uploaded immediately upon a terror event can be used to improve/optimize EMS’ decision-making regarding dispatch of evacuation resources to the scene, as information may immediately be aggregated concerning the scope, types and severities of injuries. Social media has been shown to be able to promote and support a speedier and more accurate situational awareness of emergency scenarios (Fig. 1). The current method of dispatching a ‘scouting’ team to create an initial assessment and situation reports for the medical authorities, should be re-evaluated. Critical time can be saved as the appropriate resources can be activated and directed to the scene, based on the primary situational awareness created by information relayed through social media.

Hospital personnel can also benefit from the rapid exposure to the casualties’ photos displaying specific injuries, which may facilitate preparedness to admit and treat the casualties in the medical facility. Implementing social media monitoring enable hospitals to actively collect information in order to be better informed compared to current reporting relayed by ambulance teams through conventional channels. Visual and textual updates display more actionable information that may impact on the hospital’s emergency preparations.

**Fig. 2:**
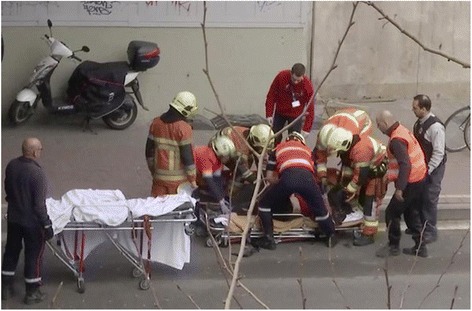
EMS personnel evacuate a casualty from the scene (taken from Twitter)

Social media may facilitate the ability to inform hospitals of the medical treatments administered to the casualties on-site (Fig. 2). To date, hospital teams receive minimal data concerning the treatment casualties receive in the field. In contrast, numerous photos of the casualties treated in the Brussels attack presented treatments provided to the casualties, at both the airport and the metro station. Active monitoring of the information disseminated through the social media, in routine and in terror events, will enable to relay such data to the EMS and hospital teams.

Considering the immediacy of information relay, it might be beneficial in unique and exceptional situations, to dispatch hospital’s medical teams to the scene. Deploying hospital staff outside their facility is generally not recommended [5], but as updated information concerning casualties can now be accessible very early, while the center of gravity is still on-site, such personnel may prove to be crucial in providing advanced life-saving medical treatments to the casualties, before and during the evacuation process. This renewed utilization of medical personnel may be especially vital in terror attacks that occur in major metropolitans, creating chaos, road blocks and a general lock-down that may result in significant traffic constraints that delay the evacuation to the hospitals.

Through bi-directional communication, EMS and hospital teams can provide real-time, crucial guidance to on-site casualties, survivors and bystanders, directing them as to how to administer initial life-saving procedures. This may improve the survivability of the victims and enable administration of vital medical care even before the arrival of EMS teams. The multi-site terror events, as occurred in Paris and Brussels, exposed a gap in resource management, and the risk of depleting medical equipment and personnel. Analyzing the time stamp of the Twitter photos from the Brussels metro station reveals that casualties waited for as long as 20 min before EMS and other emergency responders reached the scene. Real-time recruiting of civilians as first responders to provide life-saving medical actions may increase the survivability rate. Thus, empowering individuals present on-site to provide such life-saving measures, may prove to be of utmost importance. Twitter presented itself as an important communication channel, which may act as a life-line to the casualties and an asset for the responders. Twitter users who posted photos from the scenes were contacted directly, almost instantly, by journalists requesting additional information, consent to publish their photos, or interviews. Users responded in real-time and provided crucial information from the scene. Similarly, the public can be guided by medical providers to assume responsibility when formal responders are unavailable.

## Conclusions

The terror attacks that were inflicted on European cities during the last year exemplified that EMS organizations, as well as other health services, should reassess their response policies. This should include better education of the public sharing photos and information that may harm and invade patients’ privacy. There is a growing rift between the ethical norms which emergency medicine and health services’ professionals adhere to, compared to those that the public adopts while engaging in social media during emergencies. Medical guidelines and policies utilized during the response phase should be modified and adjusted to the new reality that had already changed.

The healthcare system should adopt, use and leverage social media and its advantages in designing response to terror events as well as other types of emergencies, but this requires a re-evaluation and introspection into the current emergency response models.
